# Analysis of Vestibular Labyrinthine Geometry and Variation in the Human Temporal Bone

**DOI:** 10.3389/fnins.2018.00107

**Published:** 2018-02-26

**Authors:** Lejo Johnson Chacko, Dominik T. Schmidbauer, Stephan Handschuh, Alen Reka, Karl D. Fritscher, Patrik Raudaschl, Rami Saba, Michael Handler, Peter P. Schier, Daniel Baumgarten, Natalie Fischer, Elisabeth J. Pechriggl, Erich Brenner, Romed Hoermann, Rudolf Glueckert, Anneliese Schrott-Fischer

**Affiliations:** ^1^Department of Otolaryngology, Medical University of Innsbruck, Innsbruck, Austria; ^2^Department of Biotechnology & Food Engineering, Management Center Innsbruck, Innsbruck, Austria; ^3^VetImaging, VetCore Facility for Research, University of Veterinary Medicine, Vienna, Austria; ^4^Institute of Biomedical Image Analysis, UMIT, Hall in Tirol, Austria; ^5^MED-El GmbH, Innsbruck, Austria; ^6^Institute of Electrical and Biomedical Engineering, UMIT, Hall in Tirol, Austria; ^7^Institute of Biomedical Engineering and Informatics, Technische Universität Ilmenau, Ilmenau, Germany; ^8^Division of Clinical and Functional Anatomy, Department of Anatomy, Histology and Embryology, Medical University of Innsbruck, Innsbruck, Austria; ^9^University Clinics Innsbruck, Tirol Kliniken, Innsbruck, Austria

**Keywords:** membranous labyrinth, semi-circular canals, vestibular labyrinth, microCT, centerlines, inner ear

## Abstract

Stable posture and body movement in humans is dictated by the precise functioning of the ampulla organs in the semi-circular canals. Statistical analysis of the interrelationship between bony and membranous compartments within the semi-circular canals is dependent on the visualization of soft tissue structures. Thirty-one human inner ears were prepared, post-fixed with osmium tetroxide and decalcified for soft tissue contrast enhancement. High resolution X-ray microtomography images at 15 μm voxel-size were manually segmented. This data served as templates for centerline generation and cross-sectional area extraction. Our estimates demonstrate the variability of individual specimens from averaged centerlines of both bony and membranous labyrinth. Centerline lengths and cross-sectional areas along these lines were identified from segmented data. Using centerlines weighted by the inverse squares of the cross-sectional areas, plane angles could be quantified. The fit planes indicate that the bony labyrinth resembles a Cartesian coordinate system more closely than the membranous labyrinth. A widening in the membranous labyrinth of the lateral semi-circular canal was observed in some of the specimens. Likewise, the cross-sectional areas in the perilymphatic spaces of the lateral canal differed from the other canals. For the first time we could precisely describe the geometry of the human membranous labyrinth based on a large sample size. Awareness of the variations in the canal geometry of the membranous and bony labyrinth would be a helpful reference in designing electrodes for future vestibular prosthesis and simulating fluid dynamics more precisely.

## Introduction

Balance and orientation in humans are largely dependent on the vestibular system which is housed in the temporal bone. The vestibular apparatus consists of three semi-circular canals (SCC) (posterior SCC, superior SCC, and lateral SCC) and the vestibulum that houses the otolith organs. The SCCs perceive angular acceleration using the sensory cells in the cristae ampullaris while the macular organs, namely the utricle and the saccule, perceive linear acceleration.

Movement of the SCCs activates the vestibular system generating the vestibulo-ocular reflex (VOR) which causes compensatory eye movements. The vestibular system preserves gaze stability by directing eye movements in the opposite direction to head movement. Mathematical modeling of the vestibular function has shown that SCC deflections along prime planes excite a single canal nerve, while deflections along other planes elicits maximal response where multiple canal nerves are excited (Rabbitt, 1999). Anatomical data on prime planes is mostly based on the orientation of the bony labyrinth while divergent positioning of the membranous labyrinth and inter-individual variation may influence these prime planes of highest sensitivity. One aim of this study was to quantify the variation of the alignment of bony and membranous labyrinth.

Vestibular dysfunction sometimes cannot be compensated by other senses such as vision and proprioception. That's why diseases like bilateral vestibular hypofunction (BVH) can lead to disabling oscillopsia, chronic disequilibrium, and postural instability (Ward et al., 2013). Although there are several known vestibulopathies, patients with BVH are the principal candidates for vestibular implants. Several approaches have been proposed to restore vestibular function e.g., using a multichannel vestibular prosthesis (Fridman and Della Santina, 2012). Artificial stimulation have been shown to partially restore SCC function in guinea pigs implanted with a prosthetic system (Gong and Merfeld, 2002). An electrode array implanted onto bony SCCs of Rhesus macaques was able to preserve hearing while maintaining rotational sensitivity (Rubinstein et al., 2012). Multi-channel vestibular prosthesis implants further improved vestibular function in rhesus macaques with gentamicin induced bilateral vestibulopathy (Dai et al., 2011). Directional plasticity helped restore 3D VOR in these implanted monkeys by reducing misalignments (Dai et al., 2013).

Proof of concept attempt at evoking VOR using ampullary stimulation via implanted electrodes was successful in a patient with long-term bilateral vestibular areflexia (van de Berg et al., 2012). Electrically evoked VOR (eVOR) induced by vestibular implants have also been realized to match the same frequency dependency of a normal human vestibular system (van de Berg et al., 2014). Histopathological examination has shown that multichannel vestibular prosthesis can target vestibular afferent fibers without damaging them (Sun et al., 2015).

In order to specifically target ampullar sensory organs with stimulating electrodes, substantiated knowledge about inner ear microanatomy and inter-individual variation is important. We aimed to add knowledge on human micro-anatomical geometry and inter-individual variation. Human temporal bone sections excised from cadavers have been previously examined and used to estimate inner fluid spaces (Igarashi et al., 1986). Digitized sectioned human inners have also been used to perform fluid dynamic modeling as well as to estimate the centerlines of the membranous labyrinth (Ifediba et al., 2007). Non-destructive imaging and analysis of SCC geometry and volumes were made possible by the development of high resolution computed tomography (CT) and magnetic resonance imaging (MRI) techniques. Examination and processing of the SCC geometry had previously identified an average model of the human SCC using CT scans. This study was however limited to visualize the membranous labyrinth using CT scan techniques (Bradshaw et al., 2010). Higher resolution X-ray micro tomography (μCT) of excised human temporal bone has been shown by the same group to add precise information on the membranous labyrinth (Curthoys et al., 2009).

In this work a statistical description of the human SCC geometry is provided with particular emphasis on the geometry of the membranous labyrinth. Results were obtained from 31 manually segmented μCT scans of the human inner ear. Our aim was to analyze the geometry of the membranous labyrinth in the intact human temporal bone. The geometry of the SCCs of the bony labyrinth was described previously using CT scans (Bradshaw et al., 2010). μCT scans were used to identify variations in the human cochlear microanatomy (Avci et al., 2014) and to examine the spatial orientation of the macular organs (Curthoys et al., 2009). The membranous labyrinth was examined using histological sectioning (Ifediba et al., 2007) followed by digitization. Osmium tetroxide, the routine contrasting agent for electron microscopy, enabled visualization of the membranous labyrinth like we previously showed with synchrotron radiation micro tomography (Lareida et al., 2009).

## Materials and methods

Thirty-one human temporal bones excised from donated cadavers and with no known etiologies of hearing or balance deterioration were used for this study. Human bodies were donated to the Division of Clinical and Functional Anatomy of the Medical University of Innsbruck by people who had given their informed consent prior to death for the use of their bodies for scientific and educational purposes (McHanwell et al., 2008; Riederer et al., 2012). All specimens were anonymized. Fourteen temporal bones were from the right ear and 17 temporal bones were from the left ear. The age of the subjects ranged from 6 to 90 years, average age of the specimens used was 63 (SD ± 30).

Following excision, the temporal bone specimens were fixed in 4% formaldehyde in phosphate buffer (pH 7.3) for 48 h and thoroughly washed in phosphate buffered saline (pH 7.3) (PBS). The 4% formaldehyde was always freshly prepared from paraformaldehyde and frozen at −20°C until use. The specimens were then resized carefully to meet the maximum field of view size of the microCT scanner using Luer rongeur forceps and a diamond drill bit. The resized specimens were placed in 0.1 M cacodylate buffer (pH 7.3) and placed on a shaker for several hours. The specimens were left in a 2% osmium tetroxide in 0.05 M cacodylate buffer and on a shaker at 4°C for 48 h. Osmium tetroxide post-fixation enabled visualization of the membranous labyrinth through soft tissue contrast enhancement (Lareida et al., 2009). The specimens were washed with PBS to remove all traces of unbound osmium tetroxide. The specimens were placed in a new solution of PBS and scanned in a Scanco μCT-35 scanner (Scanco Medical AG, Bruettisellen, Switzerland).

The specimens were then carefully placed in labeled plastic cassettes for embedding (cellpath, Newtown, UK) in a solution of 20% ethylene diamine tetra acetic acid (EDTA). Following which they were decalcified for a period of 6–8 weeks at 37°C in a Milestone HISTOS 5 microwave tissue processor with the EDTA solution (pH 7.4) which was replaced every week. Decalcification was performed to further increase soft tissue contrast by means of removing radiopaque mineral components. After completion of the decalcification process the specimens were thoroughly washed in PBS to remove all traces of EDTA. The specimens were left in 50% ethanol for half an hour twice and then placed in 70% ethanol. This step became necessary as the PBS solution resulted in trapped air bubbles within the labyrinth, which were in ethanol not present anymore. The specimens were scanned using an Xradia MicroXCT-400 scanner (Carl Zeiss X-ray Microscopy, Pleasanton, CA, USA). The source settings used were 45 kVp, 110 μA with angular increment between the projections set at 0.18°. The optical magnification was set at 0.4x with voxel resolution at 15 μm. Scans of the decalcified specimens were manually segmented using Amira® 6.3 visualization and analysis software (FEI Visualization Sciences Group, Mérignac Cédex, France).

### Registration and transformation

Since all the specimens were randomly oriented during scanning, all the left datasets were mirrored so that all specimens appear to be of a right inner ear. Manually segmented labels of the endolymphatic and perilymphatic spaces were merged to represent the bony labyrinth. This label was used to generate signed distance maps. The distance map of every dataset was automatically registered via mutual information metrics onto the distance map of dataset 05, which served as the reference and was chosen at random. The resulting transformation was applied to the label fields as well as to the μCT images. Additionally, small holes in the segmentation were filled (Fritscher et al., under review).

### Dataset processing in Amira®

Centerlines (bony and membranous labyrinth) and cross-sectional images (endo- and perilymphatic spaces) were generated using a semi-automated workflow (Figure S1). Segmented label data was extracted of the perilymphatic spaces, membranous labyrinth, and the entire bony labyrinth. The extracted labels were down sampled to reduce the number of unwanted branches of the centerline. The labels were then thinned and traced using Amira® (Thermo Scientific™, Hillsboro, Oregon, USA) modules (*Distance-ordered Thinner and Trace Lines*) until unbranched centerlines were evident for the bony and membranous labyrinths. Artifact branches and loops were manually removed using Amira's® *Filament editor*. All centerlines were then cut off at the same locus (Figures S2, S3) and smoothed using Amira's® *Smooth Lineset* module using standardized parameters (Smoothing −0.8, Adherence to original data 0.05, Iterations 100).

### Cross-sectional image extraction along the centerlines

**Figure 1:**
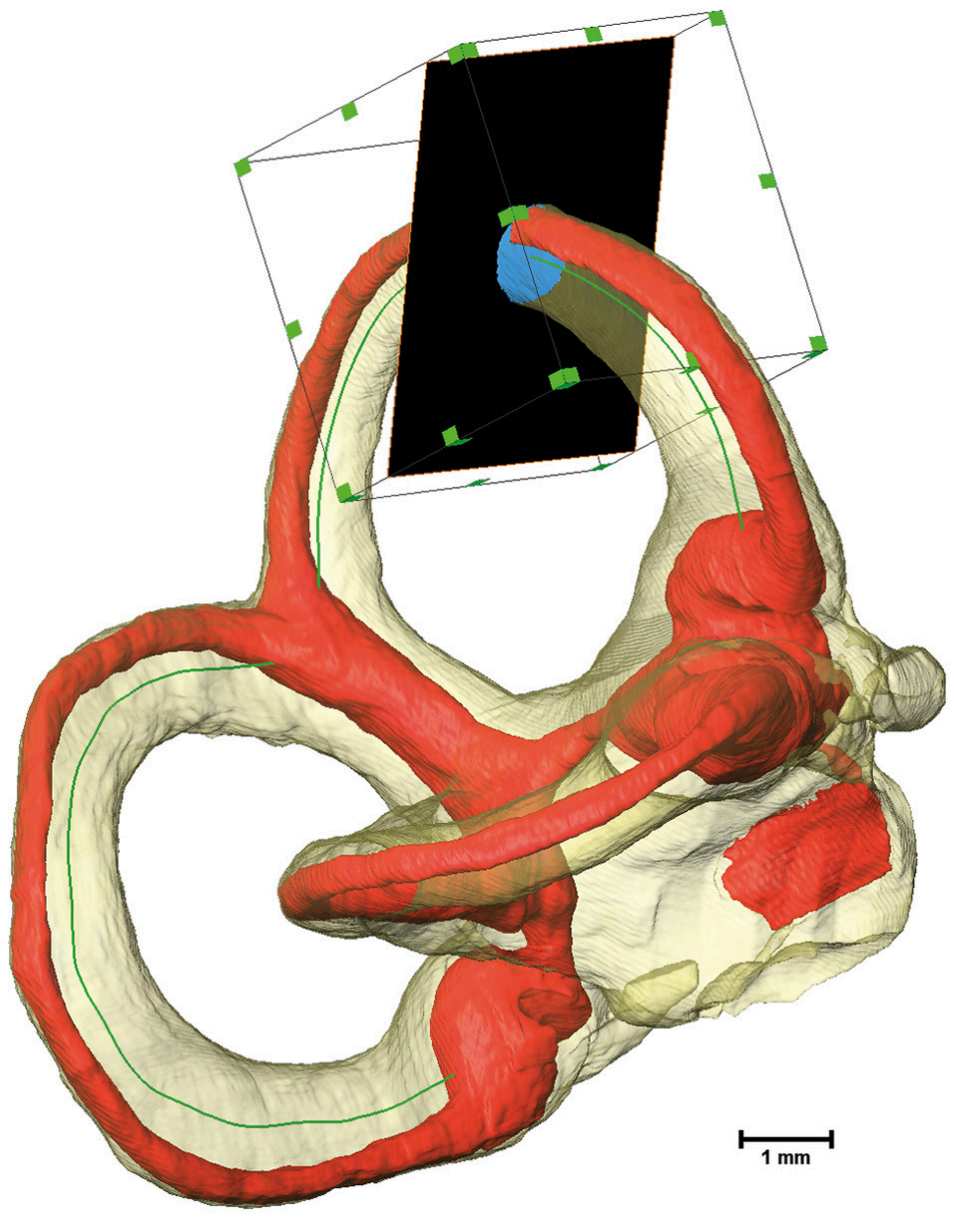
Extraction of cross-sectional images using a Region of Interest (ROI) box. The bony labyrinth is shown in transparent yellow and the membranous labyrinth in red. The green line indicates the centroid of the bony labyrinth, which serves as the trajectory for the slice, shown in black. A ROI box then crops the slice to a reasonable size.

Cross-sectional image extraction was performed using the label for the membranous labyrinth as well as for the perilymphatic spaces in combination with the actual μCT image. Two dimensional planar cross-sections were generated from three dimensional volumes using Amira's® slice module. The slice cuts the volume and associated voxels mostly in a non-orthogonal direction. The three-dimensional volume was re-sampled as a two-dimensional image. The slices were connected via a *Trajectory* module to the smoothed centerline of the bony labyrinth. The plane of the slice was defined by the point addressed by the *Trajectory* module and a vector to the next point along the centerline. As a consequence, the slice was always perpendicular to the bony labyrinth's centerline. Since the slice would cut through the whole volume, the cross-sectional area was cropped using a *Region of Interest* (ROI) box. The ROI box with an edge length of 2 mm moved along the centerline in conjunction with the slices (Figure 1).

A Tool command language (Tcl) script was implemented to move the slice and the ROI box along the designated centerline. The script connected the slice to the volumes being extracted while ensuring that the exported images (Figure S4) and all relevant data were saved separately (Supplementary Script).

### Reorientation of the segmented temporal bones

Since the segmented μCT images of the temporal bones were scanned at random orientations, they were then restored to their natural alignment in the intact human temporal bone. This was done by registering these μCT images onto a CT scan of a human temporal bone from a skull dataset. Since a comprehensive CT scan of the skull of the temporal bone donors was not available in-house, a CT scan of the human skull was downloaded from an online portal (Clark et al., 2013).

A volume rendering of the DICOM images of the human skull was created using Amira® visualizer. The rendering was then transformed to fit to Reid's baseline (Della Santina et al., 2005). Reid's baseline is defined by a plane which transects the center of each external auditory canal and also touches both the infra-orbital rims.

The bony canals of the vestibular system were then segmented by threshold segmentation. The merged labels of perilymphatic and endolymphatic compartments of dataset 05 were registered onto the label of the skull's bony labyrinth. The transformation matrix was obtained by the *getTransform* Tcl command and re-formatted as a 4 × 4 matrix. This matrix was used later on in MatLab® R2017a to restore the position and orientation within the skulls for all data-sets.

### Specimen quality assessment

**Figure 2:**
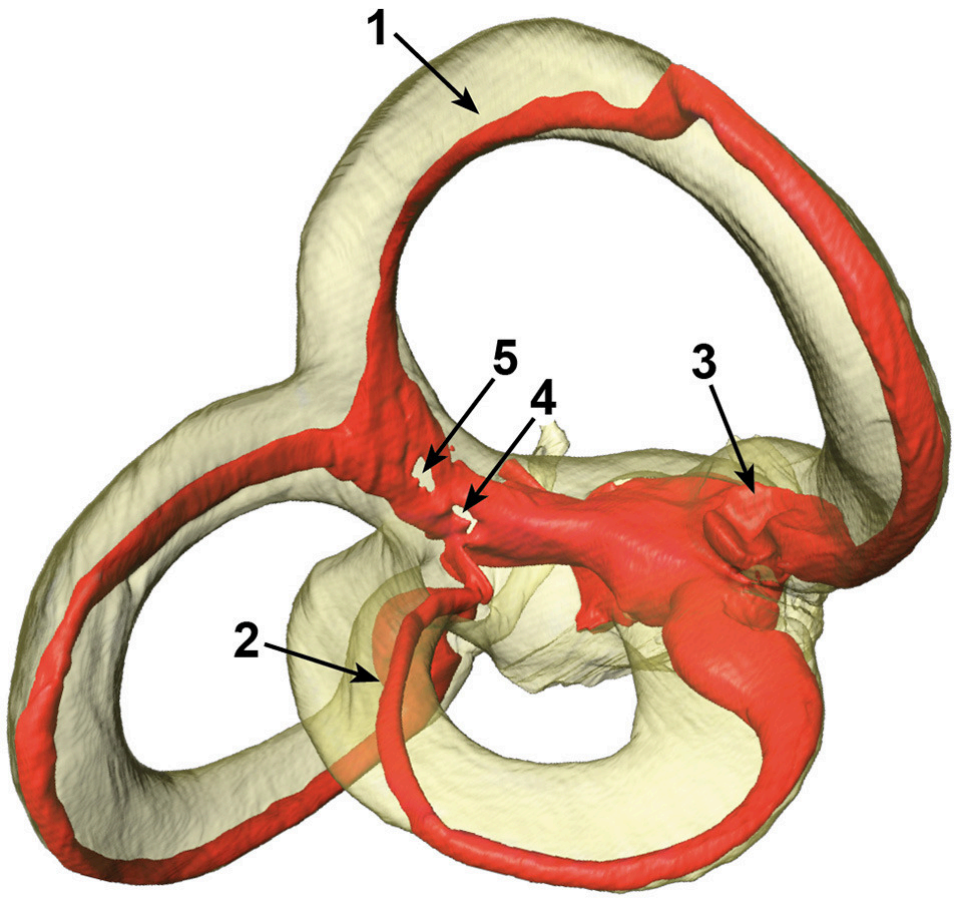
Different artifacts in the membranous labyrinth. Shown are detached superior (1) and lateral (2) membranous ducts, a partially collapsed superior ampulla (3) and a disrupted common crus (4 and 5). Segments of specimens which appeared similar where rated according to the four point scale. The posterior duct in this specimen appears to be detached in this view, but is actually just not attached to the centrifugal side of the bony canal.

The SCCs and membranous labyrinth were damaged in some of the specimens due to the surgical excision and preparation process. Quality assessment was thus done by examining renderings of the μCT images and surfaces of the labels using the Amira® software. This was possible since the surface of the bony labyrinth was rendered transparent making it easier to assess the location and appearance of the membranous labyrinth (Figure 2).

The vestibular system was subdivided into seven sections with the three SCCs, three ampullae, and the common crus under examination. The sections were then rated by three independent researchers on a four-point scale:

- 0: No obvious deviation
- 1: Membranous duct was possibly detached from the bony labyrinth
- 2: Disrupted membranous duct or collapsed ampullae
- 3: Opened or missing segment of a canal

The ratings were almost consistently throughout the researchers. Particular cases of inconsistent ratings were discussed and common consent was achieved. The ratings were applied to each individual specimen and the results were used to exclude certain sections of a specimen. The threshold selection score was set at ≤2 for the bony labyrinth and 0 for the membranous labyrinth (Table 1). This scaling system helped in inspecting only undamaged SCCs and common crus from each specimen.

**Table 1:** Scored specimens used for data analysis of the bony labyrinth and the membranous labyrinth.

	**Pos. SCC**	**Sup. SCC**	**Lat. SCC**	**Common crus**	**Pos. Amp**.	**Sup. Amp**.	**Lat. Amp**.
Bony labyrinth	26	25	29	30	–	–	–
Membranous labyrinth	24	22	23	27	26	23	26
Detached	2	3	6	1	0	1	0
Partially collapsed or disrupted	0	0	0	2	5	7	4
Cut-off or segmentation error	5	6	2	1	0	0	1

### Post-processing using MatLab® R2017a

The centerlines and the cross-sectional images were imported into MatLab® to assess the SCCs' anatomy. Artifacts in the images were removed using morphological operations. Furthermore, an algorithm was implemented to detect and exclude extracted images which showed adjacent regions e.g., at the common crus. These particular data points were excluded from further statistical estimations. Regions where these artifacts occurred have been denoted by hatched areas in the following plots. All data points were sorted and oriented in the same manner to make them comparable. The sorted and transformed data points were then interpolated for calculating average centerlines.

The centerlines' lengths were estimated by summing up the Euclidean distances between every discrete point of the lines. The distances between the centerlines of the bony and the membranous labyrinth were estimated for each point perpendicular to the centerlines by calculating the Euclidean distance.

Each cross-sectional image's area was calculated by multiplying the voxel size squared with the total number of pixels within a region. The resulting areas were further used to assess the area ratios of the membranous to the bony labyrinth.

Using a previously described method the planes were fit to each SCC's centerline (Ifediba et al., 2007). The centerline of the common crus was added to the posterior and superior SCC (Ifediba et al., 2007). Each point of the centerline was weighted by the inverse of its cross-sectional area squared using singular value decomposition (Shakarji and Srinivasan, 2013). This weighting results in planes which match the functional planes more exactly. The planes' normal vectors were then used to calculate the angles in between the SCCs of each dataset. Additionally, the angular difference of the plane fit to a SCC of the bony labyrinth and the respective plane of the membranous labyrinth was computed.

## Results

### Centerlines

**Figure 3:**
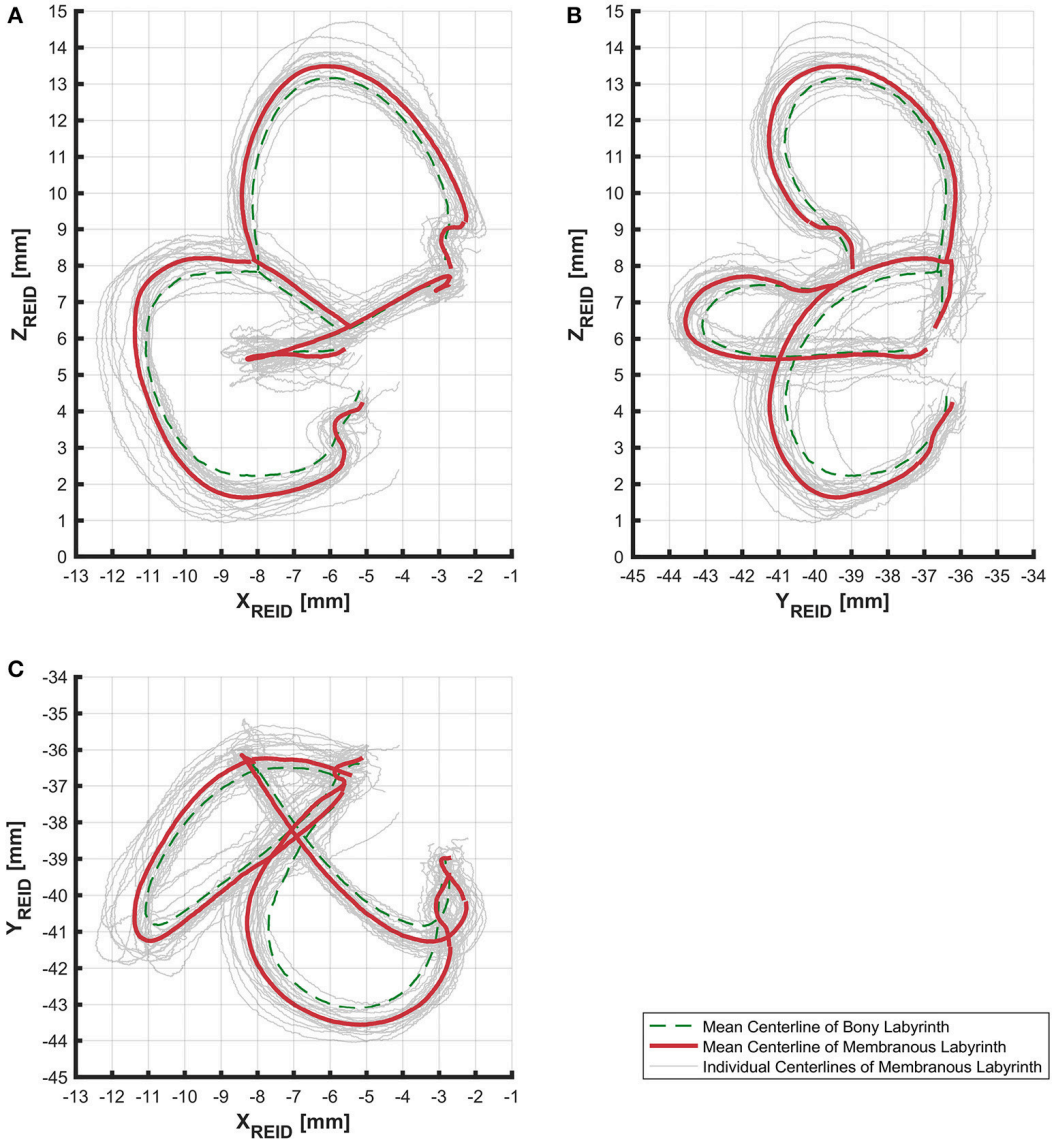
Centerlines of 31 manually segmented SCC membranous labyrinths aligned along Reid's coordinate system. Average centerlines of all the specimens in bold (red) for the membranous labyrinth and dashed lines (green) for the bony labyrinth. **(A)** Centerlines of the membranous labyrinth on the sagittal plane. **(B)** Centerlines of the membranous labyrinth on the coronal plane. **(C)** Centerlines of the membranous labyrinth on the transverse plane.

The alignment of SCCs emphasized the disparity between individual SCC centerlines of the bony (Figure S5) and membranous labyrinth (Figure 3). Individual SCC centerlines of the membranous labyrinth were aligned toward the centrifugal side of each SCC.

The lengths of the smoothed centerlines of both the bony and membranous labyrinth were measured (Table 2). The average posterior SCC centerline was longest with 16.72 mm, followed by the superior (15.05 mm) and lateral (12.57 mm) SCC the bony labyrinth. The order of lengths was the same for the average centerlines in the membranous labyrinth with 16.86 mm in the posterior, 14.94 mm in the superior, and 13.90 mm in the lateral SCC.

**Table 2:** Centerline length of bony and membranous labyrinth (All units specified in millimeters).

	**Pos. SCC**	**Sup. SCC**	**Lat. SCC**	**Common crus**	**Pos. Amp**.	**Sup. Amp**.	**Lat. Amp**.
**BONY LABYRINTH**							
Mean	16.716	15.047	12.571	2.683	–	–	–
SD	1.334	1.211	0.896	0.564	–	–	–
Min	14.939	12.898	10.997	1.303	–	–	–
Max	19.178	18.086	14.486	3.954	–	–	–
**MEMBRANOUS LABYRINTH**							
Mean	16.862	14.938	13.894	3.541	2.463	2.300	2.320
SD	1.421	1.156	1.113	0.527	0.307	0.252	0.257
Min	14.608	13.165	11.603	2.560	2.010	1.701	1.736
Max	19.530	17.432	16.242	4.879	3.054	2.770	2.835

*Centerlines were cut off at different sites according to Figures 1, 2. Note that lengths of the membranous SCCs are specified without the ampulla*.

**Figure 4:**
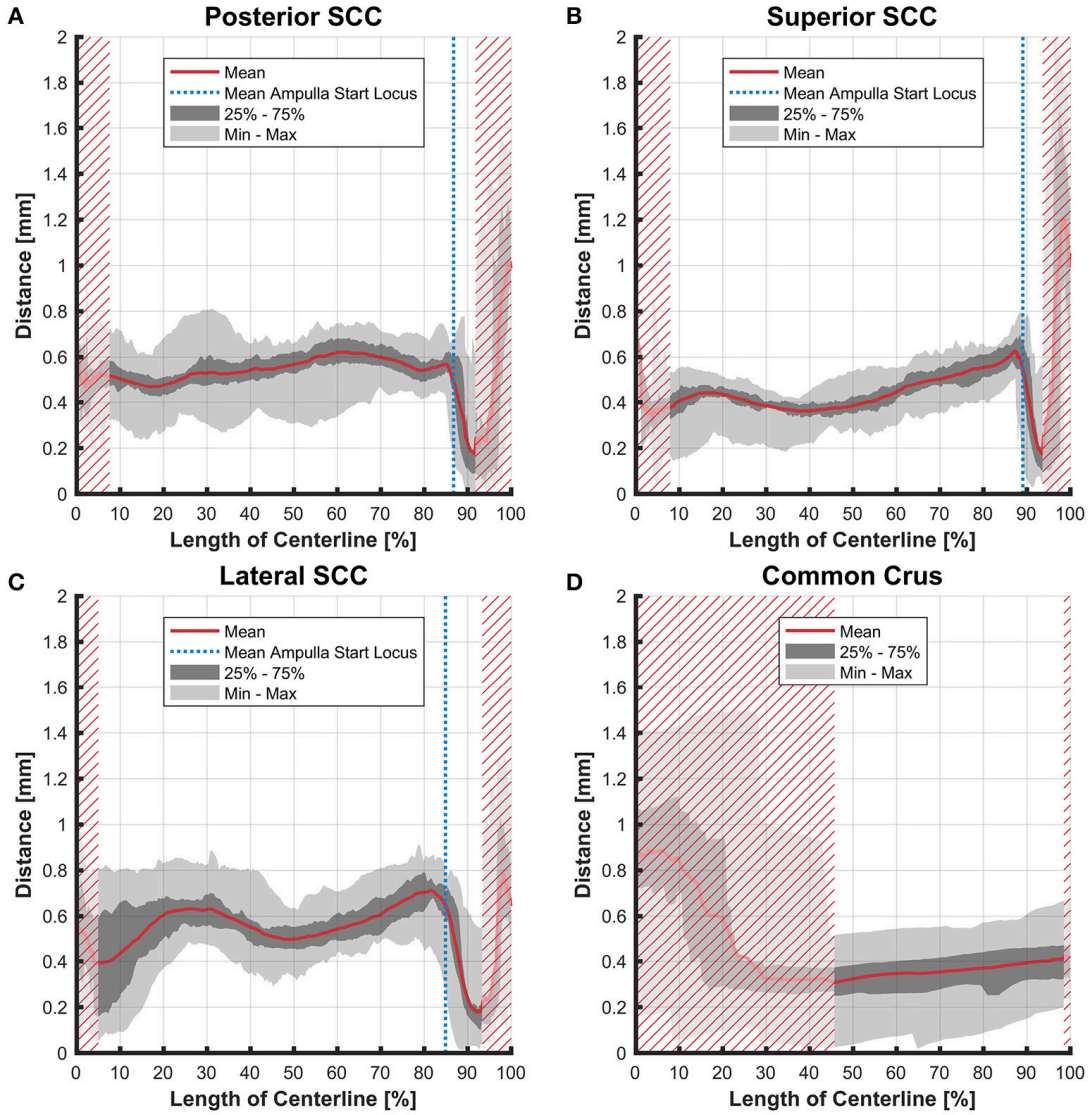
Perpendicular distances between the paths of centroids of the bony and membranous labyrinth toward the ampulla. Shown are the distances in the posterior SCC **(A)**, superior SCC **(B)**, lateral SCC **(C)**, and the common crus **(D)**. The average distance is represented by the red line (bold). Light gray areas indicate the values lying in the range of the minimum and maximum distances. The dark gray area represents the values in between the first and the third quartile. The blue line marks the mean locus where the ampulla arises. Within the red hatched area the sample size is reduced due to the presence of the regions from the adjacent SCC or ampulla.

The distance between the average centerline of the bony and the membranous labyrinth tended to be uniform for the posterior and superior SCC (Figures 4A,B). The variation was highest in the lateral SCC up to 10% centerline length (canal ending opposing the ampulla) with a range from 0.05 to 0.80 mm (Figure 4C). At the same position, the posterior and superior SCC showed only a range of 0.30–0.70 mm and 0.15–0.55 mm, respectively. High individual variation is obvious when considering the second and third quartile (0.20–0.60 vs. 0.45–0.55 mm and 0.35 and 0.45 mm, respectively).

### Cross-sectional areas

**Figure 5:**
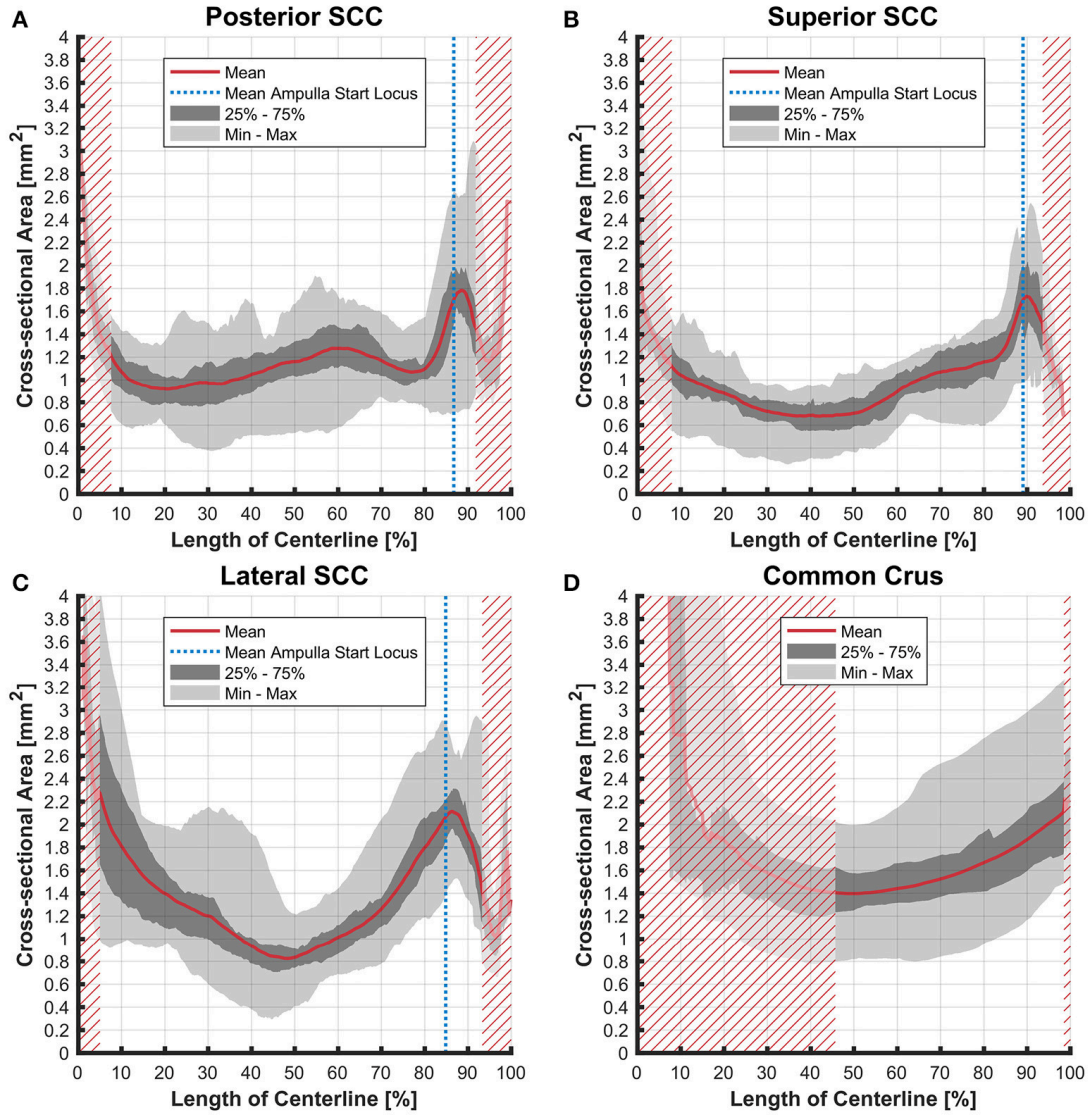
Cross-sectional area of the perilymphatic spaces stacked on the centerline of the bony labyrinth. Shown are the average cross-sectional area of the perilymphatic spaces in the posterior SCC **(A)**, superior SCC **(B)**, lateral SCC **(C)**, and the common crus **(D)**. The average area is represented by the red line (bold). Light gray areas indicate the values lying in the range of the minimum and maximum areas. The dark gray area represents the values in between the first and the third quartile. The blue line marks the mean locus where the ampulla arises. Within the red hatched area the sample size is reduced due to the presence of the regions from the adjacent SCC or ampulla.

**Figure 6:**
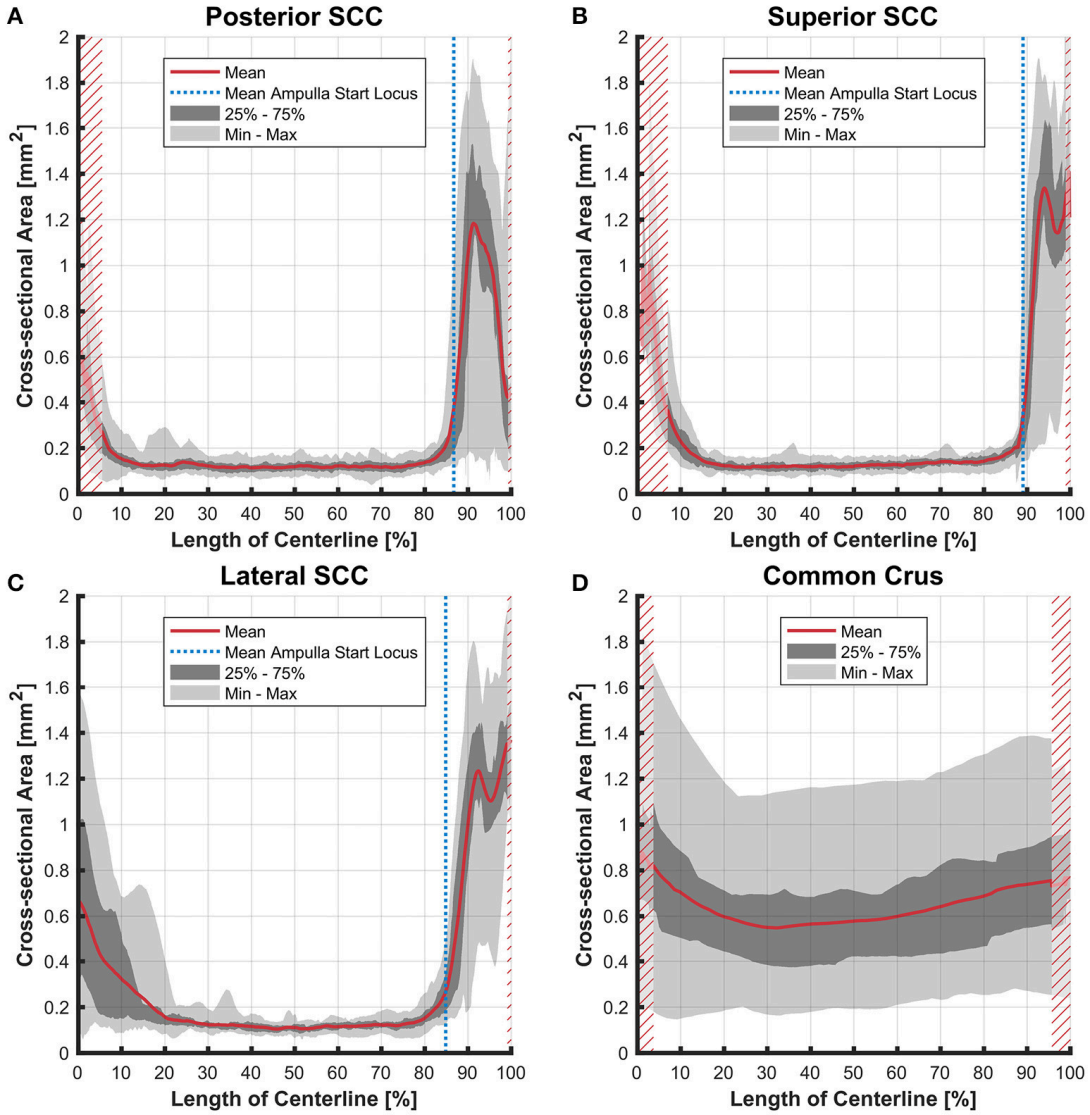
Cross-sectional area of the membranous labyrinths arrayed on the centerline of the bony labyrinth toward the ampulla. Shown are the average cross-sectional area of the membranous labyrinth in the posterior SCC **(A)**, superior SCC **(B)**, lateral SCC **(C)**, and the common crus **(D)**. The average area is represented by the red line (bold). Light gray areas indicate the values lying in the range of the minimum and maximum areas. The dark gray area represents the values in between the first and the third quartile. The blue line marks the mean locus where the ampulla arises. Within the red hatched area the sample size is reduced due to the presence of the regions from the adjacent SCC or ampulla. Note that the small dip in the red graph at ~95% centerline length represents the ampullary crest.

**Figure 7:**
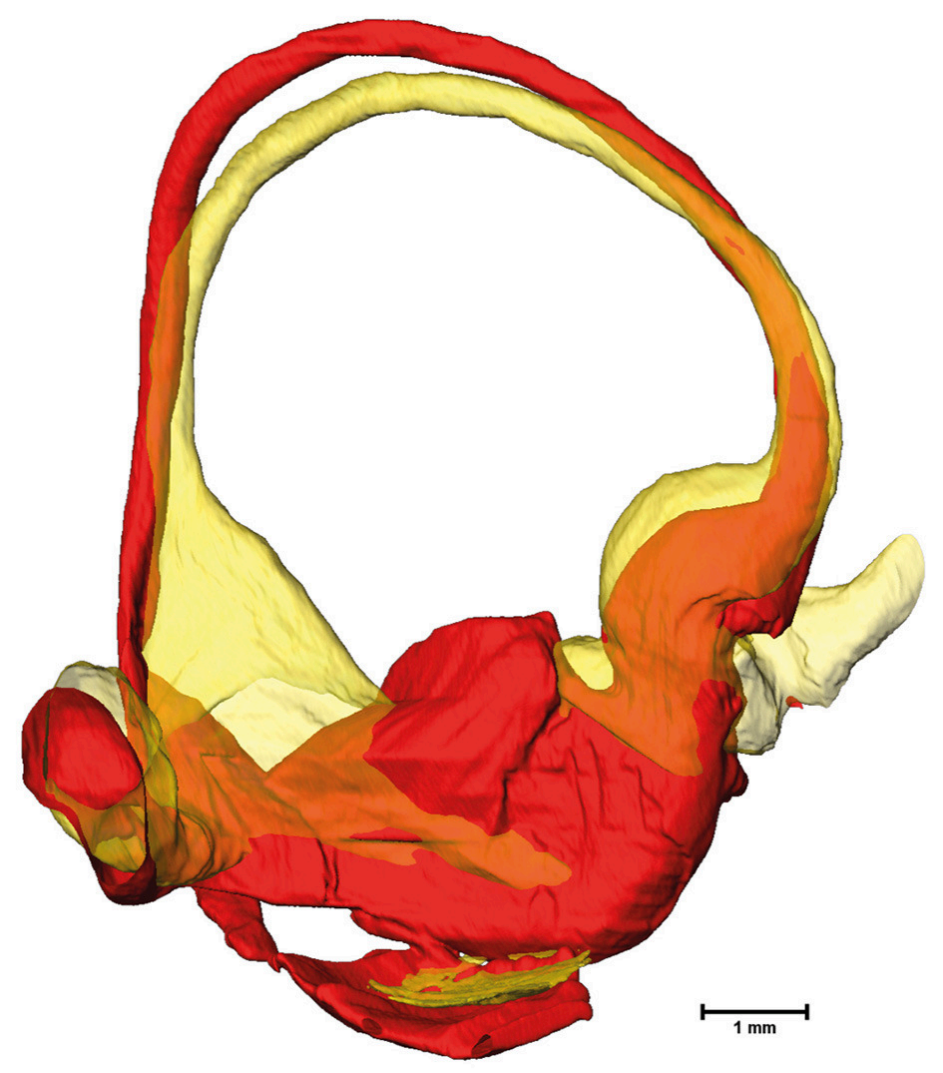
Superimposed membranous labyrinths in the lateral canal of two sample datasets. The comparison of two selected datasets (yellow and red) depicts the bulging of the SCC ending opposing the ampulla (yellow dataset) present in nearly 30% of cases.

The cross-sectional areas of the bony labyrinth (Figure S6) and perilymphatic spaces (Figure 5) were quite similar, except in the ampullary regions, where the ampullae occupy a greater portion of the bony labyrinth. Cross-sectional areas of the perilymphatic spaces in the lateral canal differ from the other two SCCs. The lateral SCC widens up toward both of its ends resulting in a parabolic course (Figure 5C). In contrast, cross-sectional areas of the SCCs' membranous labyrinth were rather uniform with cross-sectional areas ranging around 0.10 mm^2^ in the slender parts of the endolymphatic compartment (Figure 6). However, at the lateral SCC end opposing the ampulla (0–20% centerline length) we found a considerable enlargement in a subset of temporal bones (Figure 6C). This rather high inter-subject variation (0.15–1.10 mm^2^ at 5% centerline length) is also evident from the superimposed renderings representing the extreme end points (Figure 7) and was present in 29% of all the specimens examined.

### Ratios

**Figure 8:**
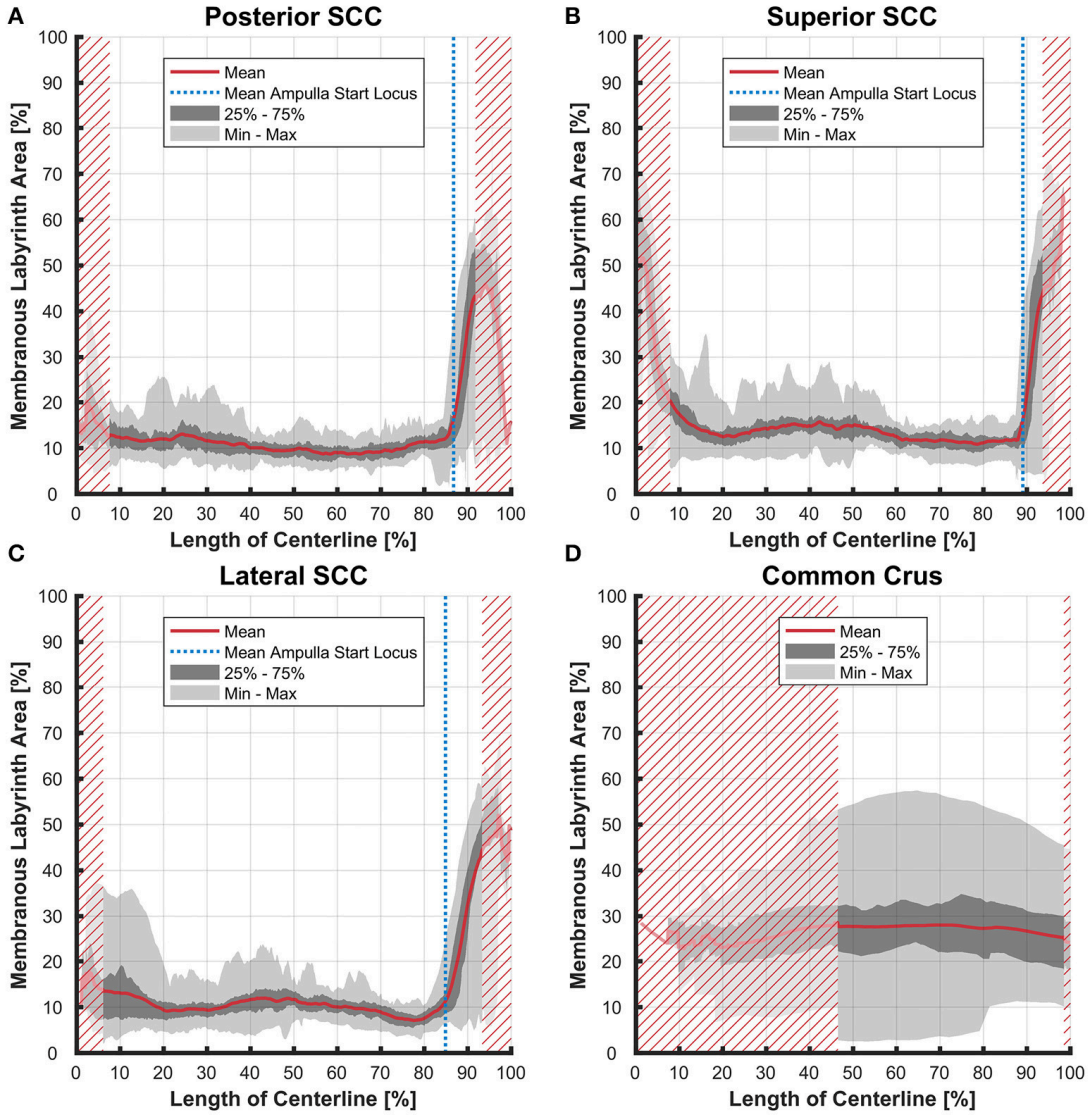
Ratio of the cross-sectional areas of the membranous labyrinth to the bony labyrinth toward the ampulla. Shown are the area ratios of the posterior SCC **(A)**, superior SCC **(B)**, lateral SCC **(C)**, and the common crus region **(D)**. The average ratio is represented by the red line (bold). Light gray areas indicate the values lying in the range of the minimum and maximum areas. The dark gray area represents the values in between the first and the third quartile. The blue line marks the mean locus where the ampulla arises. Within the red hatched area the sample size is reduced due to the presence of the regions from the adjacent SCC or ampulla. Along slender parts of SCCs only about 10% of the bony labyrinth area is occupied by the membranous labyrinth till the ampulla starts to widen.

The average ratio of the cross-sectional areas of the membranous to the bony labyrinth was between 10 and 15% in the slender parts of the membranous labyrinth (5–85% centerline length; Figures 8A,B). The exception however was in the lateral SCC (Figure 8C) where the variation was considerably higher in the region furthest away from the ampulla with a ratio ranging from 4 to 37%.

Variation in the common crus was high impeding interpretation of data on distances (0.01–1.50 mm), cross-sectional areas (0.18–1.75 mm^2^ in the endolymphatic compartment), and ratios (3–57%). Distances between the paths of centroids in the common crus were relatively small as the membranous canal is situated toward the center of the bony canal (Figure 4D). In general, cross-sectional areas of the bony labyrinth's common crus were approximately twice as high as in the SCCs (Figure S6D). The ratio of membranous to bony cross-sectional areas in the common crus is approximately twice as high as in the SCCs (Figure 8D).

### Fit plane angles

Planes were fit on the SCCs to estimate the angles between the SCCs (Table 3). These angles indicate that the bony labyrinth resembles a Cartesian coordinate system more closely than the membranous labyrinth. Posterior and superior SCCs of the bony labyrinth are almost perpendicular to each other (90.4°) while posterior—lateral and superior—lateral SCCs deviate more from being perpendicular with 87.2 and 91.8°, respectively. Average angles in the membranous labyrinth were all below 90° while superior—lateral being closest to being perpendicular (88.9°). The angles between posterior- superior and posterior—lateral were even more acute with 86.5 and 87.2°. All angles had a relatively high standard deviation of ~5°. Angles measured between posterior—superior and superior—lateral in the membranous labyrinth were 4° and 3° lower than in the bony labyrinth whereas the difference in the angle between posterior—lateral was negligible. Based on our rather large sample size, we could also demonstrate that the membranous labyrinth in the SCCs is less orthogonal than the bony labyrinth. The normal vectors and the reference points of the planes were also identified (Table S1).

**Table 3:** Average angles in between the fit planes across the various semi-circular canals.

	**Pos.—Sup**.	**Pos.—Lat**.	**Sup.—Lat**.
**BONY LABYRINTH**			
Mean	90.374	87.158	91.801
SD	4.546	5.104	4.073
**MEMBRANOUS LABYRINTH**			
Mean	86.491	87.203	88.913
SD	5.159	5.800	4.274

The fit planes were also used to estimate the angles between the membranous and bony labyrinth. The disparity in the angles tended to be the highest in the superior SCC with 4.23° (SD ± 1.56) followed by the posterior SCC with 3.37° (SD ± 1.84) and lateral SCC with 2.36° (SD ± 1.36). This difference indicates the fact that the SCCs of the bony and membranous labyrinth were not aligned exactly on the same planes.

## Discussion

The combination of higher resolution and higher contrast scans from osmified and decalcified specimens with μCT enabled a reliable visualization of the membranous labyrinth *in situ*. It also helped in avoiding artifacts from destructive processes like sectioning during histological examination. Despite the specimens belonging to different age groups and having a larger sample size, there is still considerable conformity in the SCCs. We present original data of the SCCs' membranous labyrinth cross-sectional areas. Knowledge of this data brought a higher accuracy during the plane fitting process. Previous research had only approximated membranous labyrinth data for the plane fitting process (Bradshaw et al., 2010). Compared to this study our results show a slight difference in the angles of the planes fit to the membranous labyrinth. The difference was −0.9° (posterior—superior), −2.4° (posterior—lateral), and +6.6° (superior—lateral). These results suggest a difference between the orthogonality in the bony and membranous labyrinth. We could also show that the fit planes of the membranous labyrinth SCCs deviated slightly from the planes fit on the bony labyrinth. Therefore, the approach previously used may lead to deviating plane orientations. This fact has to be taken into account, if the planes of maximum response are derived from the geometry of the bony labyrinth, e.g., via clinical CT scans.

The cross sectional areas of the SCCs of the membranous labyrinth tended to be in the same range as prescribed previously (Ifediba et al., 2007). Disparity however was observed in the lateral SCC where the average cross sectional area tended to be higher in the region furthest away from the ampulla. This may not necessarily represent an artifact from the specimen processing and could be a naturally occurring variation. Such variations would most certainly influence the fluid dynamics of the endolymph. A larger diameter was found to increase the gain but also decrease the bandwidth (Rabbitt, 1999). Whether a partial widening of the lateral SCC also affects gain and bandwidth has to be investigated using additional clinical data. Since this widening occurs in almost one third of our specimens any clinical relevance of this phenomenon has to be elucidated. From the cross-sectional areas as well as from visual inspections of the surface renderings it is evident that the isthmoidal-like narrowing in the perilymphatic spaces does not affect the geometry of the membranous labyrinth. The change of cross-sectional areas of the membranous labyrinth at the bifurcation of the common crus by a factor of six may also have an impact on fluid dynamics.

Our approach was limited by the labor intensive and time consuming segmentation process. The use of cadaveric temporal bones and tissue processing may also impact the fine structure of inner ear tissue. A very small factor of shrinkage was observed using osseous and membranous labyrinth dimensions in guinea pigs (Curthoys et al., 1977). Previously in a toad fish model, shrinkage correction factors were estimated. This was done by comparing landmark dimensions, measured in living and embedded labyrinths (Ghanem et al., 1998). The same shrinkage correction factors were later used to assess SCC morphology and functionality (David et al., 2016). A 3% shrinkage was identified in Reissner's membrane using 4% formaldehyde (Brunschwig and Salt, 1997). A significant difference in canal temporal and directional coding was absent when shrinkage correction factors were applied in the human membranous labyrinth (Ifediba et al., 2007). Due to the existence of contradictory reports we have not applied any shrinkage correction factors in our study. We assessed possible shrinkage in a parallel study describing the μCT image acquisition of these specimens (Glueckert et al., under review). We found a shrinkage factor of 1.62% through EDTA decalcification and a swelling factor of 0.55% after transferring the specimens into imaging solution of 70% ethanol for air bubble free imaging. However, shrinkage through aldehyde fixation could have an influence on electrode design and position simulations. This issue should be further addressed by more detailed investigation on the influence of aldehyde fixation on soft tissue in the human inner ear. Following preparation steps (decalcification, mild dehydration, and ethanol bathing) did not contribute much to any tissue changes after complete chemical fixation (Glueckert et al., under review).

Since our approach used an automated image extraction, adjacent SCC's were also included in the extracted images. This artifact data was however more intense at the region where the common crus meets the vestibulum and led to incorrect cross-sectional area estimation. In order to overcome this problem we implemented an algorithm which excluded affected data points. Since the temporal bones were randomly oriented during the μCT scanning process, we registered individual datasets onto the CT scan of an anonymized individual. This process might not be precise enough since the CT scan and μCT scans were sourced from different individuals. This could affect the absolute positioning of the temporal bone specimens.

We excluded designated segments after visual examination. This procedure can be justified for SCCs which were accidentally cut through preparation of the temporal bone. Deformed or partially collapsed membranous labyrinth at the ampulla organs and disrupted SCCs of the membranous labyrinth also originate most certainly from the preparation process. It can be questioned, whether detached ducts are also of unnatural origin as they might be a natural variation. Due to their appearance, we think that they were detached during preparation. For this reason, they were excluded from this study. A thorough correlative histological examination of these areas may be necessary to address this issue.

However, our data may be valuable for future vestibular electrode designs to minimize trauma to the endolymphatic compartment. Current implantation approaches involve either an intra- or extra-labyrinthine approach. The intra-labyrinthine approach in particular involves placement of an electrode within the bony canal. This ensures that electrode contacts are closest to the sensory structures with highest conductivity levels in the perilymphatic compartment. Trauma to the membranous labyrinth due to such electrode placement could result in hearing loss and should be avoided. This is due to the fact that the membranous labyrinth of the vestibular system is directly connected to the scala media in the cochlea via the small reunion duct. Both endolymphatic spaces share a common milieu comprising a high concentration of potassium ions which is indispensable for depolarizing the sensory hair cells. The electrochemical gradient present only in the cochlea may be more sensitive for potassium ion changes triggered in the vestibular system. Recently it was shown (van de Berg et al., 2017) that intra-operative electrode insertion in the human SCC is possible without acutely damaging the peripheral auditory function measured with acoustic brainstem response.

Estimation of the centerline and cross-sectional areas of the SCC's would help in electrode design and positioning. This could result in designing ideally sized electrodes which fit in the confines of the bony labyrinth while preserving the membranous structures. Assuming the electrode lead diameter used (van de Berg et al., 2017) of 0.4–0.6 mm, which results in a cross-sectional area of 0.13–0.28 mm^2^, our data support the assumption that this diameter would possibly not disrupt the membranous labyrinth. Nevertheless, it has to be considered that the cross-sectional area of the perilymphatic spaces are not shaped circularly (Figure S4). The available space for a round-shaped electrode lead is therefore slightly lower than in Figure 5. Anyhow, our normative data does not replace individual pre-operative evaluation with clinical CT and/or MRI and the evaluation of surgical techniques to safely insert electrodes. Also, any changes in the geometry of inner ear microanatomy need to be carefully screened in patients with pathological conditions.

For the first time, we were able to show statistical analysis of the SCCs' membranous labyrinth of 31 specimens from healthy individuals of different age groups.

## Author contributions

LJC: Specimen processing, contrast enhancement, manual segmentation of 31 temporal bones, evaluation of results, data interpretation. DS: Development of workflow, data processing, code writing, evaluation of results, data interpretation. SH: Temporal bone scanning and data interpretation. AR: Manual segmentation of 31 temporal bones. KF and PR: Data registration of 31 temporal bones. RS, MH, PS, and DB: Data interpretation. NF: Temporal bone processing. EP, EB, and RH: Temporal bone excision and processing. RG and AS-F: Experiment design, conception of the article, and organization.

### Conflict of interest statement

The co-author RS works as a research engineer for MED-EL GmbH in Innsbruck, Austria. The other authors declare that the research was conducted in the absence of any commercial or financial relationships that could be construed as a potential conflict of interest.

## Supplementary material

Figure S1 Flow chart of the modules used in Amira®. The labels were cropped to the size of the SCCs to reduce computing time. Arithmetic modules were used to extract the volumes of the membranous labyrinth, the perilymphatic spaces and a representation for the bony labyrinth. Volumes were then down sampled and thinned by Distance-Ordered Thinner modules. The thinned line of voxels was traced, resulting in centerlines for the bony and membranous labyrinth. After cutting the lines off and removing branches the lines were smoothed. The smoothed centerline of the bony labyrinth then served as a guideline for the slice via a Trajectory module. The slice was connected alternately to the μCT image and the volumes of the membranous labyrinth and the perilymphatic spaces. The ROI box cropped the slice to a reasonable size. Extracted images of the μCT image had to be converted before export. The sphere was used to reconstruct the position of points on the two dimensional plane in three dimensional space. Finally the Tcl Command Module applied the script (Supplementary Script).

Figure S2 Selected sites of bony labyrinth excision along the centroid of individual specimens. **(A)** Posterior centerline cut off plane; **(B)** Superior centerline cut off plane; **(C)** Lateral centerline cut off plane on the opposite side of the ampulla; **(D)** Lateral centerline cut off plane on the side of the ampulla; **(E)** Common crus cut off plane.

Figure S3 Selected sites of membranous labyrinth excision along the centroid of individual specimens. **(A)** Posterior ampulla start node; **(B)** Posterior centerline cut off plane; **(C)** Superior ampulla start node; **(D)** Superior centerline cut off plane; **(E)** Lateral centerline cut off plane on the opposite side of the ampulla; **(F)** Lateral ampulla start node; **(G)** Lateral centerline cut off plane on the side of the ampulla; **(H)** Common crus cut off plane.

Figure S4 Extracted cross-sectional images. **(A)** Cross-sectional image of the perilymphatic space; **(B)** Membranous labyrinth; **(C)**, and the actual μCT image; **(D)** Overlay of **(A–C)**.

Figure S5 Centerlines of 31 manually segmented SCC bony labyrinths aligned along Reid's coordinate system. The average centerlines of all the specimens in bold (green) for the bony labyrinth and dashed lines (red) for the membranous labyrinth. **(A)** Centerlines of the bony labyrinth on the sagittal plane. **(B)** Centerlines of the bony labyrinth on the coronal plane **(C)** Centerlines of the bony labyrinth on the transverse plane.

Figure S6 Cross-sectional area of the bony labyrinth arrayed on the centerline of the bony labyrinth. Shown are the average cross-sectional areas of the bony labyrinth in the posterior SCC **(A)**, superior SCC **(B)**, lateral SCC **(C)**, and the common crus **(D)**. The average area is represented by the red line (bold). Light gray areas indicate the values lying in the range of the minimum and maximum areas. The dark gray area represents the values in between the first and the third quartile. The blue line marks the mean locus where the ampulla arises. Within the red hatched area the sample size is reduced due to the presence of the regions from the adjacent SCC or ampulla.

Table S1 Averaged normal vectors of fit planes.

## Abbreviations

μCT: X-ray microtomography
BVH: Bilateral vestibular hypofunction
CNS: Central nervous system
CT: Computed tomography
EDTA: Ethylene diamine tetra acetic acid
eVOR: evoked Vestibulo-ocular reflex
MRI: Magnetic resonance imaging
PBS: Phosphate buffered saline
PFA: Paraformaldehyde
ROI: Region of Interest
SCC: Semi-circular canal
SD: Standard deviation
Tcl: Tool command language
VOR: Vestibulo-ocular reflex.
