# Supplementary figures and images for: Analysis of Vestibular Labyrinthine Geometry and Variation in the Human Temporal Bone
Source: Front Neurosci. 2018 Feb 26;12:107. doi: 10.3389/fnins.2018.00107 (PMC5834493; doi:10.3389/fnins.2018.00107)

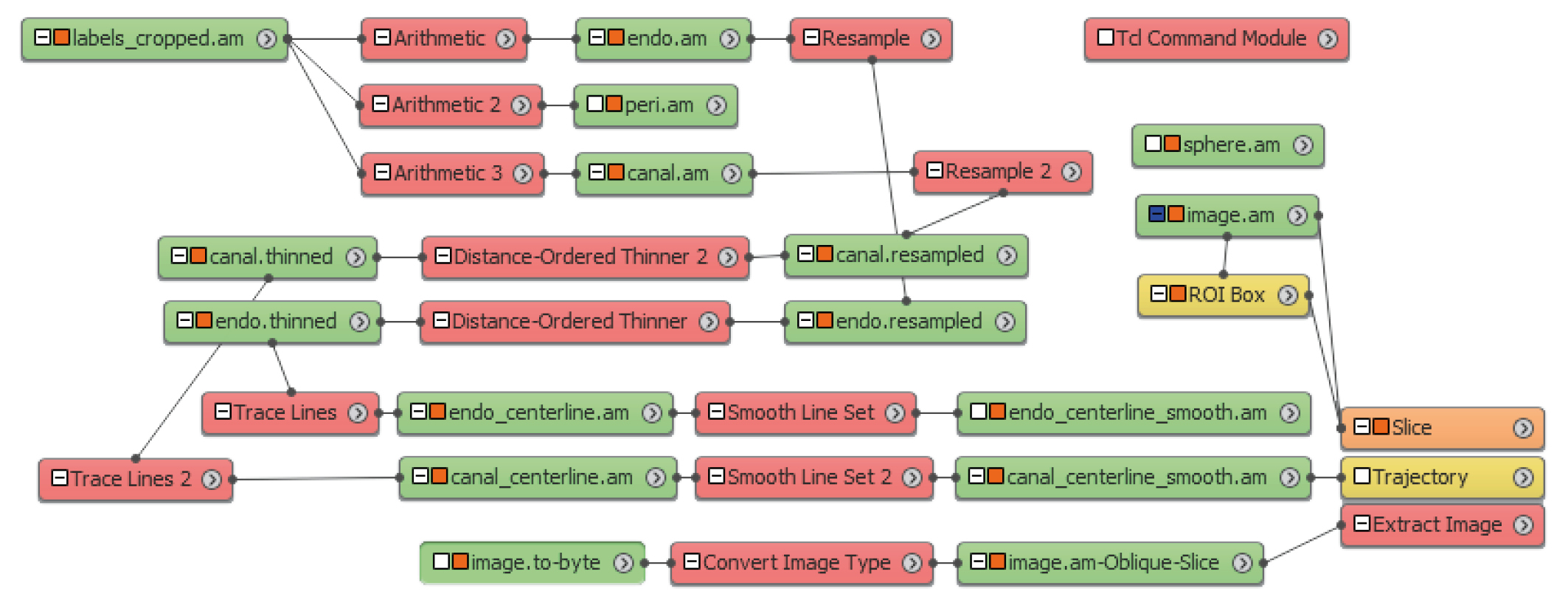

Supplement: Figure S1 — Flow chart of the modules used in Amira®. The labels were cropped to the size of the SCCs to reduce computing time. Arithmetic modules were used to extract the volumes of the membranous labyrinth, the perilymphatic spaces and a representation for the bony labyrinth. Volumes were then down sampled and thinned by Distance-Ordered Thinner modules. The thinned line of voxels was traced, resulting in centerlines for the bony and membranous labyrinth. After cutting the lines off and removing branches the lines were smoothed. The smoothed centerline of the bony labyrinth then served as a guideline for the slice via a Trajectory module. The slice was connected alternately to the μCT image and the volumes of the membranous labyrinth and the perilymphatic spaces. The ROI box cropped the slice to a reasonable size. Extracted images of the μCT image had to be converted before export. The sphere was used to reconstruct the position of points on the two dimensional plane in three dimensional space. Finally the Tcl Command Module applied the script (Supplementary Script). [file Image1.JPEG]

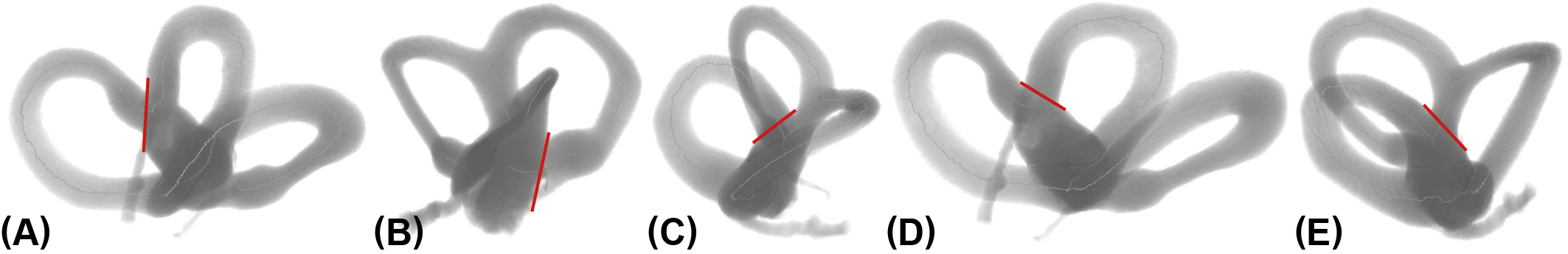

Supplement: Figure S2 — Selected sites of bony labyrinth excision along the centroid of individual specimens. (A) Posterior centerline cut off plane; (B) Superior centerline cut off plane; (C) Lateral centerline cut off plane on the opposite side of the ampulla; (D) Lateral centerline cut off plane on the side of the ampulla; (E) Common crus cut off plane. [file Image2.JPEG]

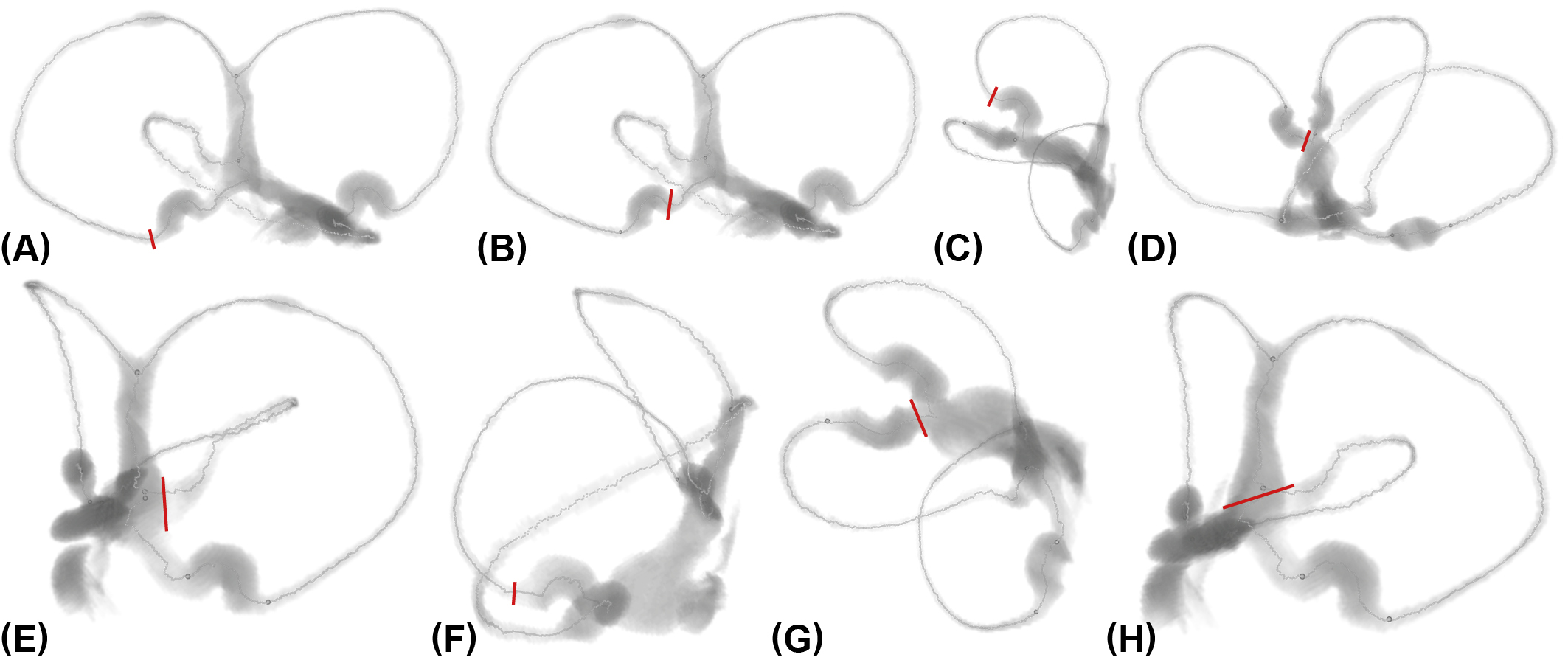

Supplement: Figure S3 — Selected sites of membranous labyrinth excision along the centroid of individual specimens. (A) Posterior ampulla start node; (B) Posterior centerline cut off plane; (C) Superior ampulla start node; (D) Superior centerline cut off plane; (E) Lateral centerline cut off plane on the opposite side of the ampulla; (F) Lateral ampulla start node; (G) Lateral centerline cut off plane on the side of the ampulla; (H) Common crus cut off plane. [file Image3.JPEG]

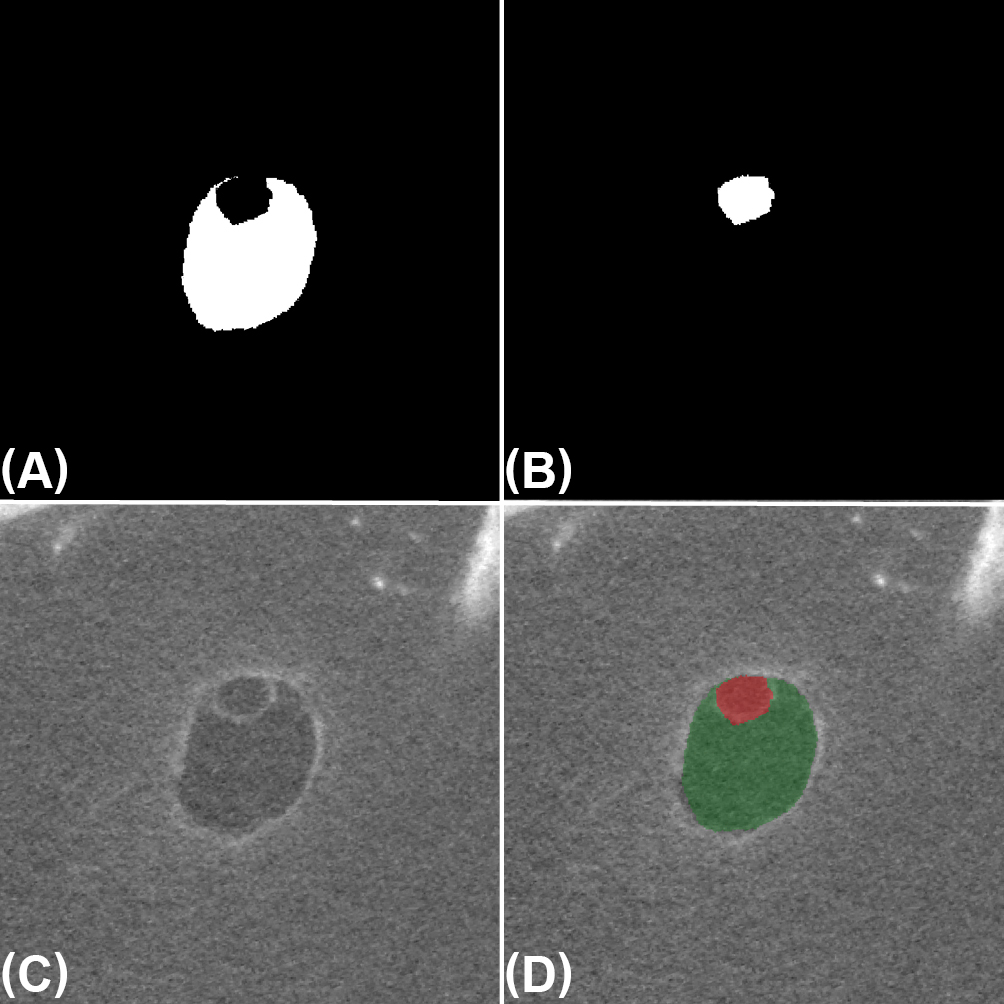

Supplement: Figure S4 — Extracted cross-sectional images. (A) Cross-sectional image of the perilymphatic space; (B) Membranous labyrinth; (C), and the actual μCT image; (D) Overlay of (A–C). [file Image4.JPEG]

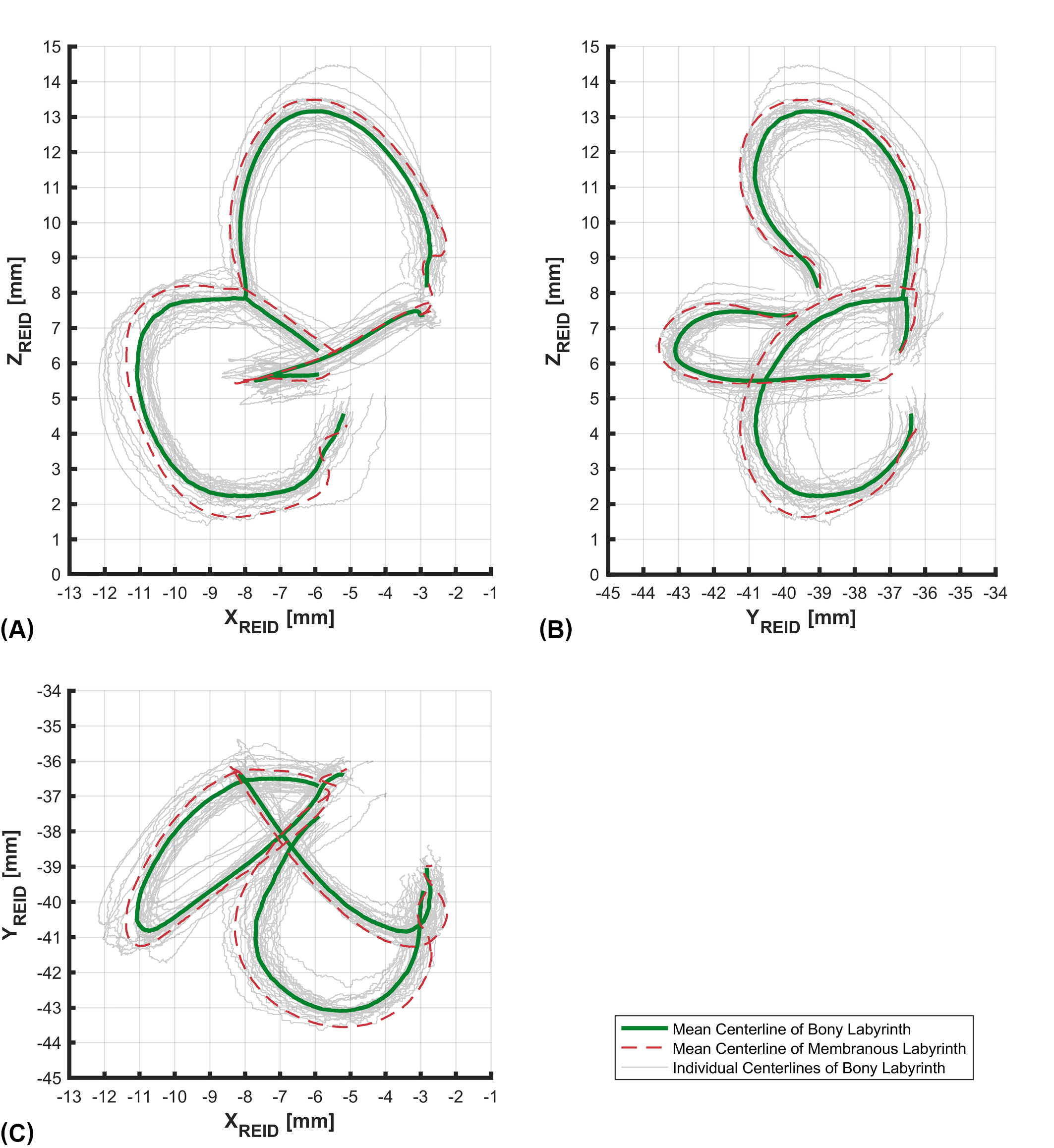

Supplement: Figure S5 — Centerlines of 31 manually segmented SCC bony labyrinths aligned along Reid's coordinate system. The average centerlines of all the specimens in bold (green) for the bony labyrinth and dashed lines (red) for the membranous labyrinth. (A) Centerlines of the bony labyrinth on the sagittal plane. (B) Centerlines of the bony labyrinth on the coronal plane (C) Centerlines of the bony labyrinth on the transverse plane. [file Image5.JPEG]

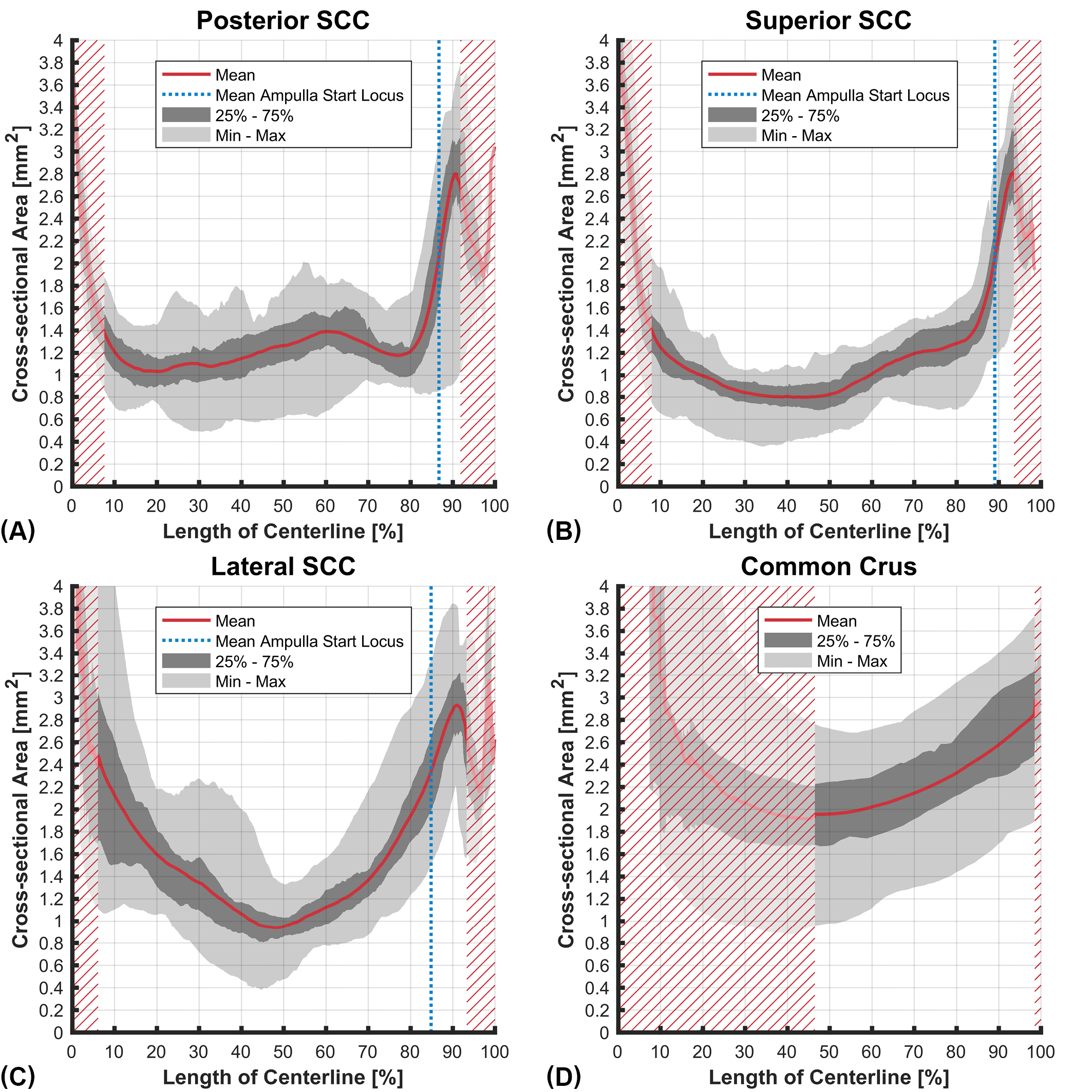

Supplement: Figure S6 — Cross-sectional area of the bony labyrinth arrayed on the centerline of the bony labyrinth. Shown are the average cross-sectional areas of the bony labyrinth in the posterior SCC (A), superior SCC (B), lateral SCC (C), and the common crus (D). The average area is represented by the red line (bold). Light gray areas indicate the values lying in the range of the minimum and maximum areas. The dark gray area represents the values in between the first and the third quartile. The blue line marks the mean locus where the ampulla arises. Within the red hatched area the sample size is reduced due to the presence of the regions from the adjacent SCC or ampulla. [file Image6.JPEG]
