# Supplementary material for: Analysis of Vestibular Labyrinthine Geometry and Variation in the Human Temporal Bone
Source: Front Neurosci. 2018 Feb 26;12:107. doi: 10.3389/fnins.2018.00107 (PMC5834493; doi:10.3389/fnins.2018.00107)
Supplement: Table S1 — Averaged normal vectors of fit planes. [file Table1.docx]

**Supplementary Table S1: Averaged normal vectors of fit planes**

|  | **Pos. SCC** |  | **Sup. SCC** |  | **Lat. SCC** |  |
| --- | --- | --- | --- | --- | --- | --- |
|  | *Mean* | *SD* | *Mean* | *SD* | *Mean* | *SD* |
| **Bony labyrinth** |  |  |  |  |  |  |
| Center |  |  |  |  |  |  |
| *X_REID_* | -9.500 | 0.410 | -6.291 | 0.174 | -6.260 | 0.348 |
| *Y_REID_* | -39.095 | 0.387 | -38.456 | 0.253 | -41.805 | 0.344 |
| *Z_REID_* | 5.036 | 0.403 | 11.834 | 0.454 | 6.033 | 0.249 |
|  |  |  |  |  |  |  |
| **Bony labyrinth** |  |  |  |  |  |  |
| Normal vector |  |  |  |  |  |  |
| *X_REID_* | -0.637 | 0.067 | -0.645 | 0.046 | 0.385 | 0.078 |
| *Y_REID_* | 0.689 | 0.045 | -0.727 | 0.038 | -0.010 | 0.072 |
| *Z_REID_* | -0.332 | 0.057 | -0.223 | 0.044 | -0.917 | 0.034 |
|  |  |  |  |  |  |  |
| **Membranous labyrinth** |  |  |  |  |  |  |
| Center |  |  |  |  |  |  |
| *X_REID_* | -9.684 | 0.392 | -6.129 | 0.276 | -6.326 | 0.385 |
| *Y_REID_* | -39.540 | 0.383 | -38.751 | 0.249 | -42.015 | 0.321 |
| *Z_REID_* | 4.515 | 0.438 | 12.044 | 0.355 | 6.089 | 0.248 |
|  |  |  |  |  |  |  |
| **Membranous labyrinth** |  |  |  |  |  |  |
| Normal vector |  |  |  |  |  |  |
| *X_REID_* | -0.658 | 0.069 | -0.650 | 0.043 | 0.382 | 0.075 |
| *Y_REID_* | 0.663 | 0.058 | -0.704 | 0.036 | 0.005 | 0.079 |
| *Z_REID_* | -0.339 | 0.065 | -0.280 | 0.036 | -0.918 | 0.031 |
