# Supplementary material for: Analysis of Vestibular Labyrinthine Geometry and Variation in the Human Temporal Bone
Source: Front Neurosci. 2018 Feb 26;12:107. doi: 10.3389/fnins.2018.00107 (PMC5834493; doi:10.3389/fnins.2018.00107)
Supplement: Supplementary file 8 [file DataSheet1.DOCX]

# Name of dataset

set DS XX

# Create folder

file mkdir T:/${DS}/endo

file mkdir T:/${DS}/peri

file mkdir T:/${DS}/sphere

file mkdir T:/${DS}/image

# Create spreadsheet and columns

set ss [create HxSpreadSheet]

$ss addColumn "Point ID" int

$ss addColumn Line int

$ss addColumn Slice int

$ss addColumn "Point 1 x" float

$ss addColumn "Point 1 y" float

$ss addColumn "Point 1 z" float

$ss addColumn "Point 2 x" float

$ss addColumn "Point 2 y" float

$ss addColumn "Point 2 z" float

$ss addColumn "Point 3 x" float

$ss addColumn "Point 3 y" float

$ss addColumn "Point 3 z" float

$ss addColumn "Voxelsize 1" float

$ss addColumn "Voxelsize 2" float

$ss addColumn "Out of Bounds" int

# Get number of lines (Value jumps to highest possible value)

Trajectory line setValue 99999

set lmax [Trajectory line getValue]

# p=point(=ID) s=slice(=Point on line) l=line(=segment) d(=Size of ROI box)

set l 0

set p 0

set d 2

## Loop through lines

while {$l <= $lmax} {

# Go to line

Trajectory line setValue $l

Trajectory fire

# Get number of points of this line (Value jumps to highest possible value)

Trajectory slice setValue 99999

set smax [Trajectory slice getValue]

set s 0

## Loop through points

while {$s <= $smax} {

# Go to slice

Trajectory slice setValue $s

Trajectory fire

# Extract coordinates of current point

set planepoint [Slice planePoint1 getValue]

set x [lindex $planepoint 0]

set y [lindex $planepoint 1]

set z [lindex $planepoint 2]

# Put ROI Box around point

"ROI Box" maximum setValues 100 100 100

"ROI Box" minimum setValues -100 -100 -100

"ROI Box" fire

"ROI Box" maximum setValues [expr $x+$d] [expr $y+$d] [expr $z+$d]

"ROI Box" minimum setValues [expr $x-$d] [expr $y-$d] [expr $z-$d]

"ROI Box" fire

# Move Sphere into bounding box

sphere.am setBoundingBox [expr $x-$d] [expr $x+$d] [expr $y-$d] [expr $y+$d] [expr $z-$d] [expr $z+$d]

sphere.am fire

# Check if ROI Box has touched global boundaries

if {[expr abs([expr ["ROI Box" maximum getValue 0]-[expr $x+$d]])]>=0.1 || [expr abs([expr ["ROI Box" maximum getValue 1]-[expr $y+$d]])]>=0.1 || [expr abs([expr ["ROI Box" maximum getValue 2]-[expr $z+$d]])]>=0.1 || [expr abs([expr ["ROI Box" minimum getValue 0]-[expr $x-$d]])]>=0.1 || [expr abs([expr ["ROI Box" minimum getValue 1]-[expr $y-$d]])]>=0.1 || [expr abs([expr ["ROI Box" minimum getValue 2]-[expr $z-$d]])]>=0.1} {

set OOB 1

} else {

set OOB 0

}

## Image export

# Connect modules to volume

Slice data connect image.am

"ROI Box" data connect image.am

# Fire Trajectory again, otherwise Slice would be in XY orientation again

if {$s==0} {

Trajectory slice setValue 1

} else {

Trajectory slice setValue 0

}

Trajectory slice setValue $s

Trajectory fire

# Fire image extraction

ExtractImage fire

# Connect image conversion module

"Convert Image Type" data connect image.am-Oblique-Slice

"Convert Image Type" fire

# Save image

image.to-byte exportData "JPEG" T:/${DS}/image/point_${p}_${l}_${s}.jpg

# Disconnect image conversion module

"Convert Image Type" data disconnect

## Sphere export

# Connect modules to volume

Slice data connect sphere.am

"ROI Box" data connect sphere.am

# Fire Trajectory again, otherwise Slice would be in XY orientation again

if {$s==0} {

Trajectory slice setValue 1

} else {

Trajectory slice setValue 0

}

Trajectory slice setValue $s

Trajectory fire

# Fire image extraction

ExtractImage fire

# Save image

sphere.am-Oblique-Slice exportData "2D TIFF" T:/${DS}/sphere/point_${p}_${l}_${s}.tif

## Endolymph export

# Connect modules to volume

Slice data connect endo.am

"ROI Box" data connect endo.am

# Fire Trajectory again, otherwise Slice would be in XY orientation again. If condition necessary for first point of each line.

if {$s==0} {

Trajectory slice setValue 1

} else {

Trajectory slice setValue 0

}

Trajectory slice setValue $s

Trajectory fire

# Fire image extraction

ExtractImage fire

# Save image

endo.am-Oblique-Slice exportData "2D TIFF" T:/${DS}/endo/point_${p}_${l}_${s}.tif

## Perilymph export

# Connect modules to volume

Slice data connect peri.am

"ROI Box" data connect peri.am

# Fire Trajectory again, otherwise Slice would be in XY orientation again

if {$s==0} {

Trajectory slice setValue 1

} else {

Trajectory slice setValue 0

}

Trajectory slice setValue $s

Trajectory fire

# Fire image extraction

ExtractImage fire

# Save image

peri.am-Oblique-Slice exportData "2D TIFF" T:/${DS}/peri/point_${p}_${l}_${s}.tif

#Get coordinates of the three points which define the slice plane

set point1 [Slice planePoint1 getValue]

set point2 [Slice planePoint2 getValue]

set point3 [Slice planePoint3 getValue]

# Save coordinates to variables

set point1x [lindex $point1 0]

set point1y [lindex $point1 1]

set point1z [lindex $point1 2]

set point2x [lindex $point2 0]

set point2y [lindex $point2 1]

set point2z [lindex $point2 2]

set point3x [lindex $point3 0]

set point3y [lindex $point3 1]

set point3z [lindex $point3 2]

# Get voxelsize of image

set voxelsize [peri.am-Oblique-Slice getVoxelSize]

# Save voxelsizes to variables

set voxelsize1 [lindex $voxelsize 0]

set voxelsize2 [lindex $voxelsize 1]

# Write values of varibles to spreadsheet

$ss setValue 0 $p $p

$ss setValue 1 $p $l

$ss setValue 2 $p $s

$ss setValue 3 $p $point1x

$ss setValue 4 $p $point1y

$ss setValue 5 $p $point1z

$ss setValue 6 $p $point2x

$ss setValue 7 $p $point2y

$ss setValue 8 $p $point2z

$ss setValue 9 $p $point3x

$ss setValue 10 $p $point3y

$ss setValue 11 $p $point3z

$ss setValue 12 $p $voxelsize1

$ss setValue 13 $p $voxelsize2

$ss setValue 14 $p $OOB

# Increase variables

incr p

incr s

}

incr l

}

# Save spreadsheet

$ss exportData "Microsoft XML Spreadsheet 2003" T:/${DS}/imagedata.xml

endo_centerline.am exportXML T:/${DS}/endo_centerline.xml

endo_centerline_smooth.am exportXML T:/${DS}/endo_centerline_smooth.xml

canal_centerline.am exportXML T:/${DS}/canal_centerline.xml

canal_centerline_smooth.am exportXML T:/${DS}/canal_centerline_smooth.xml
